# Effect of Vertical and Lateral Offset Restoration on Clinical Outcomes in Intracapsular and Extracapsular Hip Fractures Undergoing Hemiarthroplasty

**DOI:** 10.7759/cureus.22617

**Published:** 2022-02-26

**Authors:** Recep Dincer, Anil Gulcu, Atay Tolga, Özgür Başal, Ahmet Aslan, Yakup B Baykal

**Affiliations:** 1 Orthopedics and Traumatology, Suleyman Demirel University Faculty of Medicine, Isparta, TUR; 2 Orthopedics and Traumatology, Alanya Alaaddin Keykubat University, Faculty of Medicine, Antalya, TUR; 3 Orthopedics and Traumatology, Emsey Hospital, Istanbul, TUR

**Keywords:** lateral offset, femoral offset, leg length discrepancy, hemiarthroplasty, calcar replacement

## Abstract

Objective

We aimed to investigate whether there is a change in the postoperative lateral and vertical femoral offset (FO) in patients who underwent bipolar straight stem hemiarthroplasty (SSHA) and calcar stem hemiarthroplasty (CRHA) and whether this change makes a difference in the comparison of both groups.

Material and methods

This study included 109 patients who met these criteria. Patients are divided into two groups according to treatment methods. There were 58 patients (group 1) who underwent SSHA due to intracapsular (AO type 31-B neck and 31-C head fracture) femur fracture, and there were 51 patients (group 2) who underwent CRHA due to extracapsular (AO type 31-A intertrochanteric) femur fracture. We analyzed femoral vertical and lateral femoral offset, Wiberg angle, and head-neck angle difference in both groups.

Results

The median age was significantly higher in the CRHA group (p=0.042). The Harris hip score (HHS) was significantly higher in the SSHA group (p=0.023). The femoral offset difference was 5 mm in the SSHA group, while it was significantly lower (-6 mm) in the CRHA group (p<0.001). The Wiberg angle difference did not differ significantly between patient groups (p=0.214). The limb length difference was found to be similar in both surgical groups (p=0.483).

Conclusions

The study results show that there was no negative correlation between clinical and radiological outcomes in the SSHA group, whereas there was a negative correlation between clinical and radiological outcomes in the CRHA group. It is very difficult to control vertical and lateral offset reconstruction, especially in extracapsular hip fractures reconstructed by hemiarthroplasty. Deficiencies in lateral and vertical stabilization restoration may be associated with poor clinical outcomes in CRHA patients. Orthopedic surgeries should be performed carefully when restoring leg length and femoral offset, especially calcar replacement hemiarthroplasties.

## Introduction

Intracapsular and extracapsular hip fractures due to low-energy trauma are one of the most important causes of functional failure, morbidity, and mortality in elderly patients [1-3]. Various methods such as internal or external fixation and arthroplasty are available for the surgical treatment of these fractures. Strategies are being developed to improve surgical technique and fixation devices in these fragile patients with multiple comorbidities [4-6]. Hemiarthroplasty is an appropriate treatment option for selected patients as it provides early mobilization and good functional results in unstable intertrochanteric and femoral neck fractures [2,3,7,8].

Although studies in the literature have mainly focused on perioperative mortality and complications following hemiarthroplasty, the number of studies investigating the postoperative functional results has also been increasing in recent years [8-10]. There is current evidence to suggest that offset plays a role in patient pain and function following total hip arthroplasty (THA) [9-13]. Similarly, postoperative clinical outcomes after bipolar hemiarthroplasty, daily living activity, and quality of life may be affected by the change of the lateral offset [14,15]. The biomechanical results of offset in hemiarthroplasty are scarcely compared with THA in the literature [10,14]. There are a wide variety of prostheses in use, with varying degrees of modularity and offsets [13]. Unlike the modular stem, traditional femoral stems do not offer options for variable offset. Therefore, it is not always possible to restore preoperative vertical and horizontal offset [15]. The functional effect of length and offset restoration after hemiarthroplasty is less clear. Only a few studies addressed this question with no clear conclusion [14,16,17]. The limitation of implant variety and disruption of the anatomy creates difficulties and affects postoperative function. To the best of our knowledge, to date, in English literature, there have been no studies presenting lateral and vertical femoral offset (FO) results in patients who underwent bipolar hemiarthroplasty with straight stem for intracapsular and calcar replacement stem for extracapsular hip fractures.

Our objectives in this study are as follows: to investigate whether there is a change in the postoperative lateral and vertical femoral offset in patients who underwent hemiarthroplasty due to intracapsular and extracapsular hip fractures and to investigate whether there is a relationship between lateral and vertical femoral offset change and clinical outcomes.

## Materials and methods

This retrospective study was conducted after the approval of the Suleyman Demirel University Faculty of Medicine Clinical Research Ethics Committee (12/3/2020-52593). For the study, the medical recorded data of 393 patients who underwent bipolar hemiarthroplasty with straight and calcar replacement stem due to intracapsular and extracapsular hip fracture in Orthopedics and Traumatology Clinic between January 2013 and December 2017 were reviewed. The study was conducted in accordance with the principles of the Declaration of Helsinki. Written informed consent was obtained from each patient included in this study.

**Figure 1:**
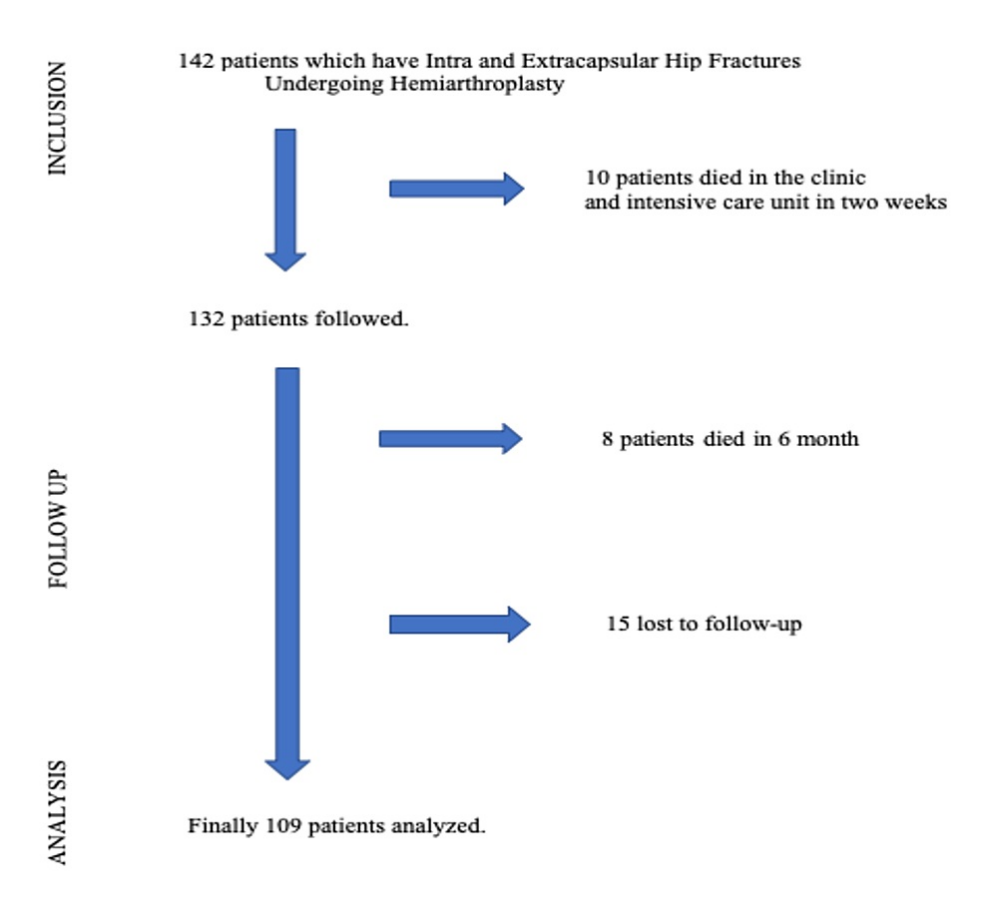
Flow diagram for patients included

The inclusion criterion was patients over 60 years of age who underwent bipolar hemiarthroplasty. Calcar replacement stem prostheses (CRHA) were selected for extracapsular fractures with very unstable trochanter minor fractures with low-energy trauma and patients unable to undergo closed reduction and internal fixation. Straight stem hemiarthroplasty (SSHA) was chosen for intracapsular fractures, with a follow-up of at least one year. Patients treated with internal fixation and hemiarthroplasty for revision of failed internal fixation; patients with fractures with dysplastic hips (instead of congenital growth hormone deficiency associated with hip dysplasia); patients with cognitive disorders such as dementia, Alzheimer’s disease, delirium, or psychiatric disease; patients younger than 60 years; those who were lost to follow-up and who exhibited pathological fractures; those with polytrauma with concomitant fractures; those with incomplete medical records; and patients who died during the first year of the postoperative period were excluded from this study. This study included 109 patients who met these criteria (Figure 1).

All patients were operated on by two different surgical teams. A posterolateral incision was used in all patients in the lateral decubitus position. Cemented prostheses were used in 109 patients with the first-generation cementing technique. Straight stem (Bipolar, Ortopro Orthopedics, Beykoz, Istanbul, Turkey) and calcar stem (Tıpmed, Bornova, İzmir, Turkey) were used. The size of the head of the prosthesis was the same as the size of the femoral head removed intraoperatively from the patient. If there was no femoral head with the same diameter, the suitable head size was used to be 1 mm depending on the vacuum effect. The correct implant diameter and position were determined by intraoperative fluoroscopy. A postoperative routine protocol was implemented. After 24 hours, drains of the patient were removed. On postoperative day 1, mobilization was achieved with the help of physiotherapists by applying full weight-bearing on the pain tolerance.

Outcome measurements

The primary outcome measurements were lateral and vertical femoral offset (FO) in patients who underwent hemiarthroplasty due to intracapsular and extracapsular hip fractures. The change in postoperative lateral and vertical FO in the fractured hip compared to the intact side was investigated, and other parameters (femoral neck, Wiberg angle, etc.) were measured on postoperative radiographs. The secondary outcomes measured at the last follow-up were clinical results using the Harris hip score (HHS) and pain by visual analog scale (VAS) at the last follow-up [18,19].

Demographic characteristics including age, gender, side, American Society of Anesthesiologists (ASA) physical status score [20], anesthesia type, operation time, and length of stay in hospital were collected from medical records. Postoperative complications were noted. Patients included in the study were analyzed retrospectively and divided into two groups according to treatment methods. There were 58 patients in group 1 who underwent SSHA due to intracapsular (AO type 31-B and 31-C) fracture. There were 51 patients who underwent CRHA due to extracapsular (AO type 31-A2 intertrochanteric femur) fracture.

**Figure 2:**
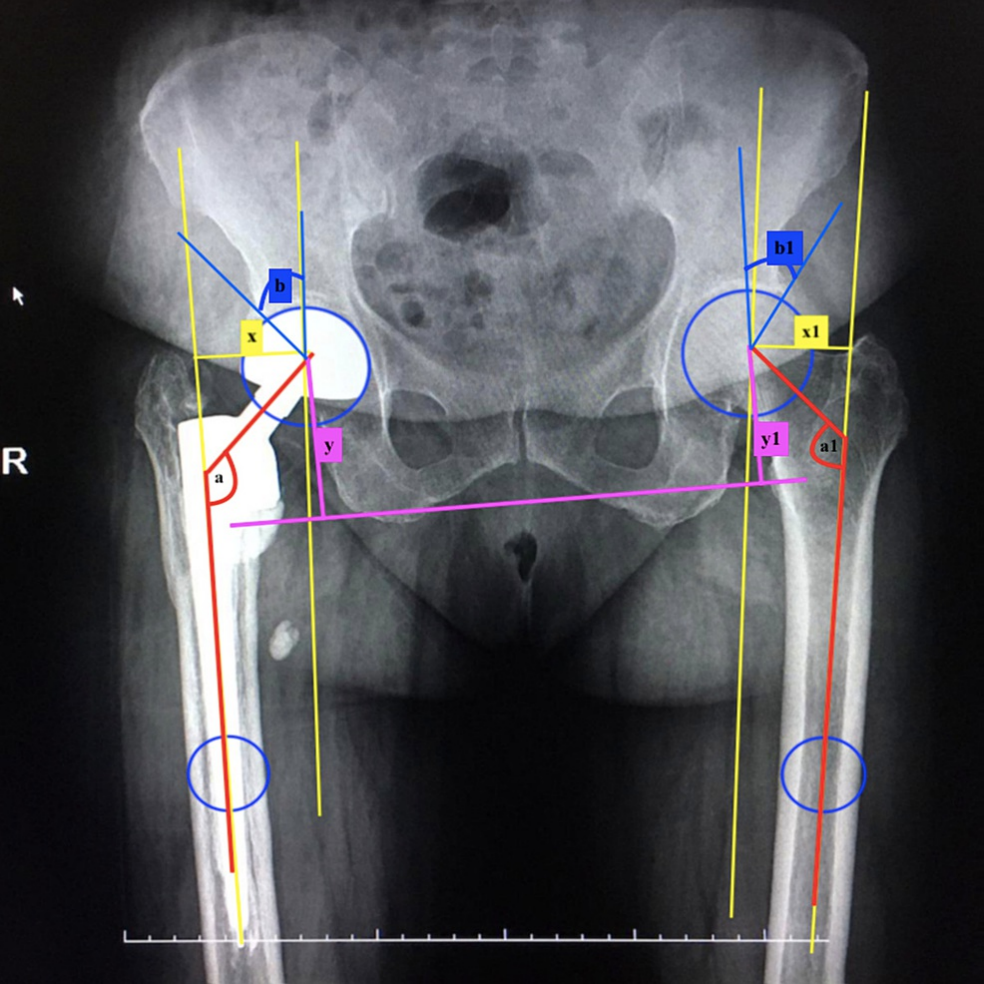
Anatomical measurements of the calcar stem hemiarthroplasty side and intact side values Anatomical measurements were evaluated by calculating the difference between the calcar stem hemiarthroplasty side and intact side values. The femoral neck offset difference is the horizontal distance between the hip rotation center and the femoral shaft anatomical axle difference between the prosthetic side and the intact side (yellow lines: x-x1). The femoral vertical offset difference was measured as the vertical distance between the center of the femoral head and the trochanter minor between the prosthetic side and intact side (pink lines: y-y1). The head-neck angle difference is the difference between the partial prosthesis and the stem (red lines: a-a1). The center edge angle of the Wiberg angle is the perpendicular line drawn to the center of the head and the lateral corner of the acetabulum roof difference between the prosthetic side and the intact side (blue lines: b-b1). The distance between the trochanter major and the lateral corner of the acetabulum roof difference between the prosthetic side and the intact side (green line: z-z1).

**Figure 3:**
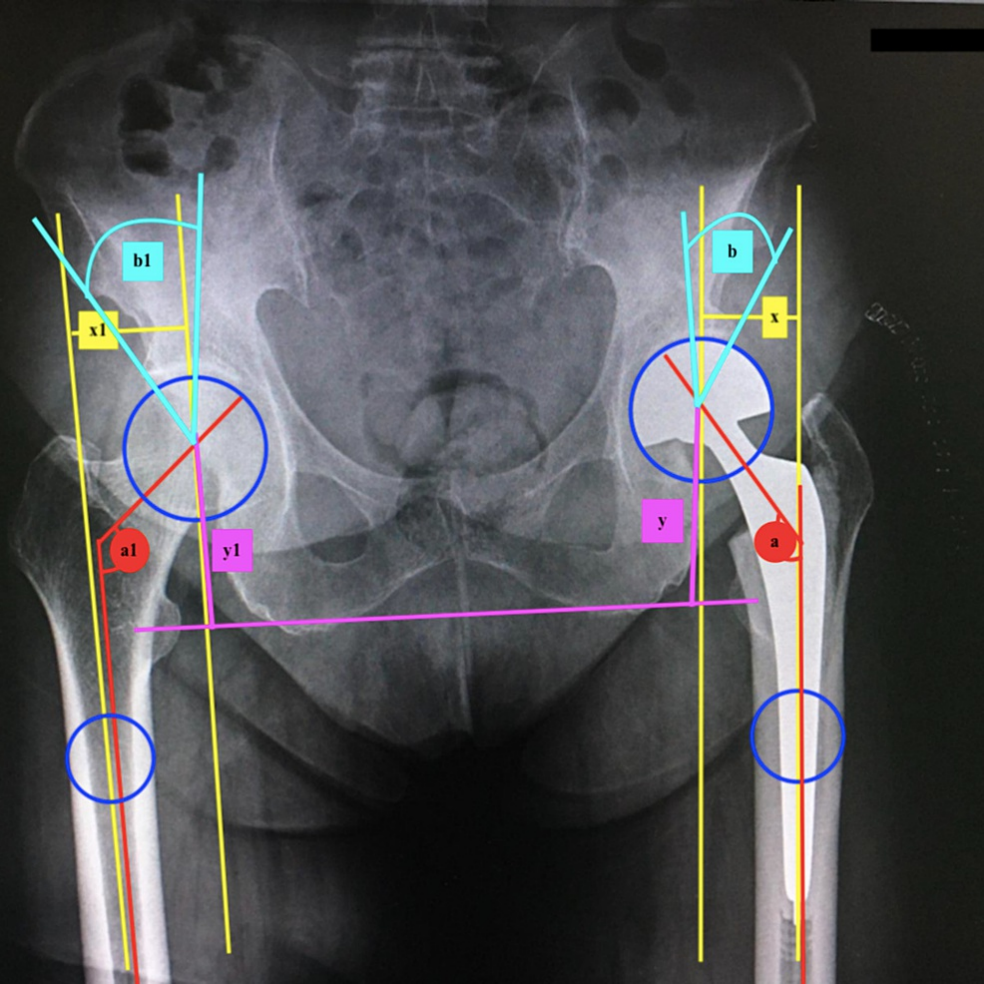
Anatomical measurements of the straight stem hemiarthroplasty side and intact side values Anatomical measurements were evaluated by calculating the difference between the straight stem hemiarthroplasty side and intact side values. The femoral neck offset difference is the horizontal distance between the hip rotation center and the femoral shaft anatomical axe difference between the prosthetic side and the intact side (yellow lines: x-x1). The femoral vertical offset difference was measured as the vertical distance between the center of the femoral head and the trochanter minor between the prosthetic side and the intact side (pink lines: y-y1). The head-neck angle difference is the difference between the partial prosthesis and the stem (red lines: a-a1). The center edge angle of the Wiberg angle is the perpendicular line drawn to the center of the head and the lateral corner of the acetabulum roof difference between the prosthetic side and the intact side (light blue lines: b-b1).

Radiological images were acquired, and radiological parameters were measured. Anteroposterior (AP) pelvis digital radiography was performed in all patients on postoperative day 2 according to the standard protocol. Pelvis AP radiography was performed in the supine position, with the limbs at 10°-15° of internal rotation and the pubic symphysis in the center. Two different physician observers who were blinded to the approach used performed all radiological measurements [13,21]. Radiography measurements were performed using ENLIL PACS (Eroğlu Information Technologies, Eskisehir, Turkey) and standardized with other healthy hips: femoral lateral offset difference was measured as the horizontal distance between hip rotation center and femoral shaft anatomical axle, femoral vertical offset difference was measured as the vertical distance between the center of the femoral head and the center of the trochanter minor, and head-neck angle difference was obtained by measuring the angle of the neck of the partial prosthesis and stem. The Wiberg angle difference was measured as the angle between the perpendicular line drawn to the center of the prosthetic head and the lateral corner of the acetabulum roof. Anatomical measurements were evaluated by calculating the difference between the prosthetic side and intact side values (Figures 2, 3).

Statistical analysis

SPSS version 20.00 statistical software (IBM Corporation, Armonk, NY, USA) was used for statistical analysis. Categorical data was given as frequency (percentage), and normal distribution of quantitative data was given with mean and standard deviation. Variables without normal distribution data were given as median (minimum-maximum). The normality of the continuous data was tested using the Kolmogorov-Smirnov test. Two-group comparisons were performed using the Mann-Whitney U-test for variables without normal distribution. A Chi-square test was used for the statistical comparisons of categorical variables, and descriptive statistics were expressed as frequency (%). The Spearman correlation test was used for the analysis of the relationship between numerical variables. P-values of <0.05 were considered significant.

## Results

The demographic data of the patients according to groups are presented in Table 1. There was no statistically significant difference between the groups in terms of demographic findings such as gender and side (p=0.195 and p=0.679, respectively). However, the median age in the CRHA group (84) was found to be significantly higher than in the SSHA group (81) (p=0.042).

**Table 1:** Demographic data of group 1 and group 2 patients *: Mann–Whitney U-test, **: Chi-square test

	Group 1	Group 2	P-value
Age, median (minimum-maximum)	81 (65–92)	84 (67–94)	0.042*
Sex, n (%)			0.195**
Female	41 (70.7%)	30 (58.8%)	
Male	17 (29.3%)	21 (41.2%)	
Side, n (%)			0.679**
Right	33 (56.9%)	27 (52.9%)	
Left	25 (43.1%)	24 (47.1%)	

No statistically significant difference was found between the groups in terms of the patient’s perioperative characteristics such as operation time, length of stay in the hospital, follow-up periods, ASA score, and type of anesthesia (p=0.120, p=0.277, p=0.881, p=0.860, and p=0.797, respectively) (Table 2). On the other hand, when compared in terms of complications (infection, periprosthetic fracture, and dislocation), there was no difference between the two groups (p=0.212, p=0.871, and p=0.484, respectively) (Table 2).

**Table 2:** Preoperative, intraoperative, and postoperative features and complications of group 1 and group 2 LOS: length of stay in the hospital, number of infections: total count, *: Mann–Whitney U-test, **: Chi-square test

	Group 1	Group 2	p-value
Operation time (minute), median (minimum-maximum)	48 (40–72)	50 (45–75)	0.120*
LOS (day), median (minimum-maximum)	8 (2–20)	9 (3–21)	0.277*
Follow-up (month), median (minimum-maximum)	40 (30–61)	39 (28–60)	0.881*
ASA score, n (%)			0.860**
II	31 (53.4%)	26 (51%)	
III	22 (37.9%)	19 (37.3%)	
IV	5 (8.6%)	6 (11.8%)	
Anesthesia type, n (%)			0.797**
Spinal	31 (53.4%)	26 (51.%)	
General	27 (46.6%)	25 (49%)	
Infection, n (%)			0.212**
+	3 (5.2%)	6 (11.8%)	
-	55 (94.8%)	45 (88.2%)	
Periprosthetic fracture, n (%)			0.871**
+	3 (5.2%)	3 (5.9%)	
-	55 (94.8%)	48 (94.1%)	
Dislocation, n (%)			0.484**
+	1 (1.7%)	2 (3.9%)	
-	57 ( 98.3%)	49 (96.1%)	

The median VAS scores were 4 (1-7) in the SSHA group and 4 (1-8) in the CRHA group. There was no significant difference in VAS scores between the two groups (p=0.390) (Table 3). The median HHS was 79 (43-94) in the SSHA group and 68 (39-91) in the CRHA group. There was a significant difference in HHS between the two groups (p=0.023) (Table 3).

**Table 3:** Evaluation of the differences (between prosthetic and intact hip) of anatomical measurements of patients undergoing straight and calcar stem hemiarthroplasty LLD: leg length discrepancy, HHS: Harris hip score, VAS: visual analog scale, *: Mann–Whitney U-test

	Straight stem hemiarthroplasty, median (minimum,maximum)	Calcar stem hemiarthroplasty, median (minimum,maximum)	p-value^*^
Femoral lateral offset (mm)	5 (-6,7)	-6 (-10,9)	<0.001
Femoral neck angle (X°)	5 (2,9)	7 (3,11)	0.001
Wiberg angle (x°)	3 (-7,8)	2 (-11,9)	0.214
LLD (mm)	2 (-7,9)	3 (-12,13)	0.483
HHS	79 (43,94)	68 (39,91)	0.023
VAS	4 (1,7)	4 (1,8)	0.390

As stated below, the comparison of all perioperative radiological measurement outcomes is presented in Table 3. While the median femoral head-neck angle was 5° in the SSHA group, it was 7° in the CRHA group (p=0.001). The median femoral offset difference was 5 mm in the SSHA group, while it was significantly lower (-6 mm) in the CRHA group (p<0.001). The Wiberg angle difference did not differ significantly between patient groups (p=0.214). Similarly, no significant statistical difference was found between the two groups in terms of the median leg length differences (p=483). In the correlation analysis performed, a low level of negative correlation (rho=-0.268; p=0.042) was observed between the Harris hip score and VAS scores in the SSHA group. However, there was no found correlation between HHS and other radiological measurements, such as femoral offset, leg length discrepancy, femoral neck, and Wiberg angle, in the SSHA group (Table 4).

**Table 4:** Correlation between functional outcome and biomechanical restoration in straight stem hemiarthroplasty patients rho: Spearman correlation coefficient, n: total straight hemiarthroplasty patients, HHS: Harris hip score, LLD: leg length discrepancy, *: Spearman’s correlation analysis

n (58)	rho	P-value*
HHS and VAS	-0.268	0.042
HHS and femoral offset	0.029	0.826
HHS and LLD	-0.213	0.108
HHS and femoral neck angle	-0.011	0.932
HHS and Wiberg angle	-0.244	0.065

There was a moderate negative correlation between HHS and femoral head-neck angle difference (rho=-0.484; p<0.001) and VAS (rho=-0.441; p=0.001) in the CRHA group (Table 5). Also, there was a weak correlation between HHS and the lateral femoral offset value (r =-0.301, p=0.032), but there was a significant relationship. There was a weak correlation between HHS and the leg length differences, but there was a significant relationship (r =-0.284, p=0.043). However, there was no found correlation between the HHS and the Wiberg angle in the CRHA group (Table 5).

**Table 5:** Correlation between functional outcome and biomechanical restoration in calcar stem hemiarthroplasty patients rho: Spearman correlation coefficient, n: total calcar hemiarthroplasty patients, HHS: Harris hip score, LLD: leg length discrepancy, *: Spearman’s correlation analysis

n (51)	rho	P-value*
HHS and VAS	-0.441	0.001
HHS and femoral offset	-0.301	0.032
HHS and LLD	-0.284	0.043
HHS and femoral neck angle	-0.484	<0.001
HHS and Wiberg angle	0.118	0.411

## Discussion

The present study demonstrates the impact of femoral offset on anatomical measurements and clinical functional results in patients who underwent SSHA and CRHA. The main outcome of this study, in terms of clinical outcomes, is that it shows that HHS scores are significantly better in the SSHA group than in the CRHA group. In terms of radiological results, the femoral head-neck angle difference and femoral lateral offset difference were found to be significantly worse in the CRHA group than in the SSHA group. When the correlation of clinical and radiological results is analyzed, in the CRHA group, a moderately strong negative correlation was found between HHS, femoral head-neck angle, and VAS. In addition, there was a negative correlation even a little between the HHS and the lateral femoral offset and LLD in the CRHA group. There was a weak relationship between HHS and only VAS in the SSHA group, but no relationship was found with other parameters. Another result of this study that should be noted is that the mean age in the CRHA group was found to be significantly higher than that in the SSHA group. This difference may be due to the higher incidence of intertrochanteric fractures in older ages [22].

FO is responsible for providing the biomechanical tension of abductor muscles [23]. Studies on the biomechanical results of offset about hemiarthroplasty are few in the literature, and these studies are generally associated with the results of hemiarthroplasty for displaced femoral neck fractures [13-17]. In this current study, only a low level of negative correlation was observed between the Harris hip score and the VAS scores in the SSHA group, whereas there was a moderate negative correlation between the HHS and the femoral head-neck angle difference in the CRHA group. Also, in the CRHA group, there was a weak correlation between HHS and the lateral femoral offset value, but there was a significant relationship. There was a weak correlation between the HHS and the leg length differences, but there was a significant relationship.

A number of factors may be related to our results. Perhaps the most important of these is that we could not find any study related to the clinical results of hemiarthroplasty due to intertrochanteric fracture and lateral and/or vertical femoral offset. Studies have also indicated that high femoral offset does not always lead to good results. The authors concluded that high offset would increase the length of the abductor arm, thereby increasing the tension in the abductor muscles and the iliotibial system, which could cause more pain [14,23,24]. In this study, while the median femoral head-neck angle was 5° in the SSHA group, it was 7° in the CRHA group. The median femoral offset difference was 5 mm in the SSHA group, while it was significantly lower (-6 mm) in the CRHA group. There was a moderate negative correlation between the HHS and the femoral head-neck angle difference and VAS in the CRHA group. Also, there was a weak correlation between the HHS and the lateral femoral offset value, but there was a significant relationship.

Vertical femoral offset problems or LLD is often seen after hip arthroplasty, and it has been reported that it can cause back pain, discomfort, instability, and limping, especially when the difference exceeds 1.5 cm [20,25]. The reduction in FO can cause lengthening of the limbs in response to soft tissue tension. Therefore, an attempt is made to restore the patients’ natural FO by selecting a standard or high offset femoral stem [26]. In our study, there was even a little negative correlation between the HHS and the lateral femoral offset and LLD in the CRHA group. However, there was no found correlation between the HHS and FO and LLD in the SSHA group. Important complications associated with hemiarthroplasties, such as dislocation, infection, aseptic loosening, and periprosthetic fracture, have been reported [27]. However, previous studies have mainly focused on dislocations, mainly associated with complications of hemiarthroplasty. With more detailed examination of hip biomechanics and the development of implant technologies, dislocation and revision rates are decreasing, contrary to the popular belief [14,17,28]. Mukka et al. reported that the dislocation rate for bipolar cement prostheses in 373 patients in whom the posterolateral approach was used was 10.7% [28]. In the present study, dislocation was detected in three (2.8%) of the 109 patients. We believe that this is due to the correct capsule repair rather than providing good hip anatomy suitable for biomechanics [29]. Moreover, in our study, no statistically significant difference was found between the groups in terms of infection, periprosthetic fracture, and dislocation.

Limitations and strengths

The retrospective nature of the study and absence of gait analysis are the limitations of the present study. The standard treatment of all patients and comprehensive characterization of the cohort provide interpretability and comparability for future studies. This study may be the first study in the literature that evaluated the anatomical femoral offset values of straight stem hemiarthroplasty and analyzed the offset functional outcomes in patients who underwent hemiarthroplasty with calcar replacement.

## Conclusions

The study results show that there was no negative correlation between clinical and radiological outcomes in the SSHA group, whereas there was a negative correlation between clinical and radiological outcomes in the CRHA group. It is very difficult to control vertical and lateral offset reconstruction, especially in extracapsular hip fractures reconstructed by hemiarthroplasty. Deficiencies in lateral and vertical stabilization restoration may be associated with poor clinical outcomes in CRHA patients. Orthopedic surgeries should be performed carefully when restoring leg length and femoral offset, especially calcar replacement hemiarthroplasties.
